# Osteogenesis Imperfecta: A Case Series and Literature Review

**DOI:** 10.7759/cureus.33864

**Published:** 2023-01-17

**Authors:** Constanza Neri Morales, Alejandra Silva Amaro, José D Cardona, Joanna L Bendeck, Karen Cifuentes Gaitan, Valentina Ferrer Valencia, María T Domínguez, María L Quevedo, Isabel Fernández, Luis G Celis Regalado

**Affiliations:** 1 Department of Biosciences, University of La Sabana, Chía, COL; 2 Department of Radiology, University Hospital Fundación Santa Fe de Bogotá, Bogotá, COL; 3 Department of Pediatrics, St. Christopher’s Hospital for Children, Philadelphia, USA; 4 Department of Medical Genetic Unit, Caracas Metropolitan Polyclinic, Caracas, VEN

**Keywords:** type i osteogenesis imperfecta, type ii osteogenesis imperfecta, type iii osteogenesis imperfecta, common mutation, pediatric genetics, osteoarticular disorders, skeletal dysplasia

## Abstract

Osteogenesis imperfecta (OI) is a hereditary disease of connective tissue characterized by the loss of bone density and mass, which increases the fragility of the bones, thus presenting multiple fractures throughout the years followed by bone deformity and articular instability. This condition has various clinical presentations. We present four cases of OI, one case with type I, two cases with type II, and one case with type III. The clinical diagnosis in most of the cases was clinical; only one of them was confirmed with genomic sequence. The treatment of these cases was based on medication, orthopedic surgery, and recovery and physical therapy. The evolution was torpid in these cases but with prolonged life expectancy despite the severity and type of OI. It is important to highlight that the patients did not have a neurocognitive compromise. Early diagnosis and multidisciplinary medical management are crucial in obtaining better outcomes for this disease, improving the quality of life, and avoiding complications.

## Introduction

Osteogenesis imperfecta (OI) is a hereditary disease of connective tissue characterized by the loss of bone density and mass that increases the fragility of the bones, thus presenting multiple fractures throughout the years followed by bone deformity and articular instability. This condition has various clinical presentations such as dentinogenesis imperfecta, blue sclerae, short stature, hearing loss developed through time, and cardiac malformations [1,2]. In 90% of cases, OI is an autosomal dominant genetic disease, and in the other 10%, it is idiopathic, where mutations are found in *COL1A1* or *COL1A2* genes, these being the ones that encode the α1 and α2 chain of type I collagen. The incidence of OI is one in 15,000-20,000 live births with a 10 in 100,000 prevalence, affecting both sexes equally, without preference for any race or ethnic group [2-4].

Due to the high heterogeneity of this condition, it presents great variability in its clinical presentation. The first classification of OI was carried out by Sillence, where it was divided into four types; later, other types and subtypes were added due to the different mutations, arriving at a total of 18 types. The diagnosis of OI is mainly clinical and can be done pre- or postnatally [5-7].

We present four cases of OI, one of type I, two of type II, and one of type III. The diagnosis in most cases was clinical and in two of them prenatally. Only one of them was confirmed with genomic sequencing. The treatment of these cases was based on medication, orthopedic surgery, and recovery and physiotherapy. The evolution was torpid with multiple fractures and deformities in the skeletal system in these cases but with a prolonged life expectancy despite the severity and type of OI.

## Case presentation

Case 1

A male patient product of the second pregnancy of a mother with a history of OI was delivered via cesarean at 28 weeks, weighing 1,245 g. At birth, the patient went bradycardic with poor respiratory effort and required orotracheal intubation. Four days later, the patient presented edema and tenderness in the right lower extremity, which turned out to be a fracture as evidenced by an X-ray.

**Figure 1:**
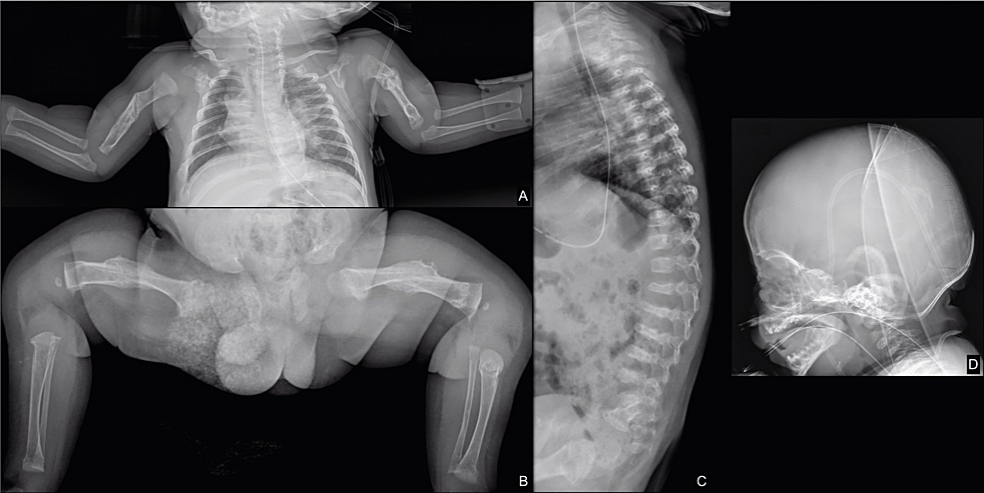
Inpatient follow-up imaging. (A) Upper and (B) lower limb X-rays at three years old showing several previous fractures in the long bones, the mid-shaft of both femurs, and the humeri with callus formation. Previous right rib and bilateral metaphyseal fractures were also identified, particularly in the lower limbs. (C) Lateral spine X-ray demonstrating multiple flattened, mid-thoracic and lumbar, vertebral bodies (platyspondyly). (D) Lateral skull X-ray with evidence of deficient mineralization and thinning of the cranial vault with small ossification centers, particularly in the parietal region.

The patient had multiple X-rays during the course of his disease. In the four-year follow-up, long bone X-ray imaging detected severe healing fractures, in both upper and lower limbs, some of them demonstrating residual angular deformity. One of the fractures, in the mid-shaft of the left humerus, was acute (Figure 1). Imaging findings and family history of OI were considered, and assessment by orthopedics and genetics was ordered.

Phenotype

The clinical genetics service assessed the newborn, and during the physical examination, they reported a normocephalic patient, normotensive fontanelle, normal implantation of the ears, blue sclerae, and short neck.

Genotype

Massive gene sequencing for OI including *COL1A1*, *COL1A2*, *CRTAP*, *FKBP10*, *P3H*, *PLOD2*, *PPIB*, *SERPINF1*, *SP7*, *CRTAP*, *FKBP10*, and *ANO5* was ordered. Two gene variants are identified *COL1A2* heterozygous NM_000089.3, CHR7: G.94429291G>A. C.3815G>A; P.CYS1272TYR and *COL1A1* heterozygous NM_000088.3, CHR17: G.50194441C>T. C.1522G>A; P.ALA508THR.

Due to all these findings, they suggested a clinical case compatible with type III OI. Specialized care to minimize the risk of fractures and continued regular follow-up with pediatric orthopedics, pediatrics, and genetics services were suggested. At this moment, the patient is six years old with normal neurocognitive development.

Case 2

**Figure 2:**
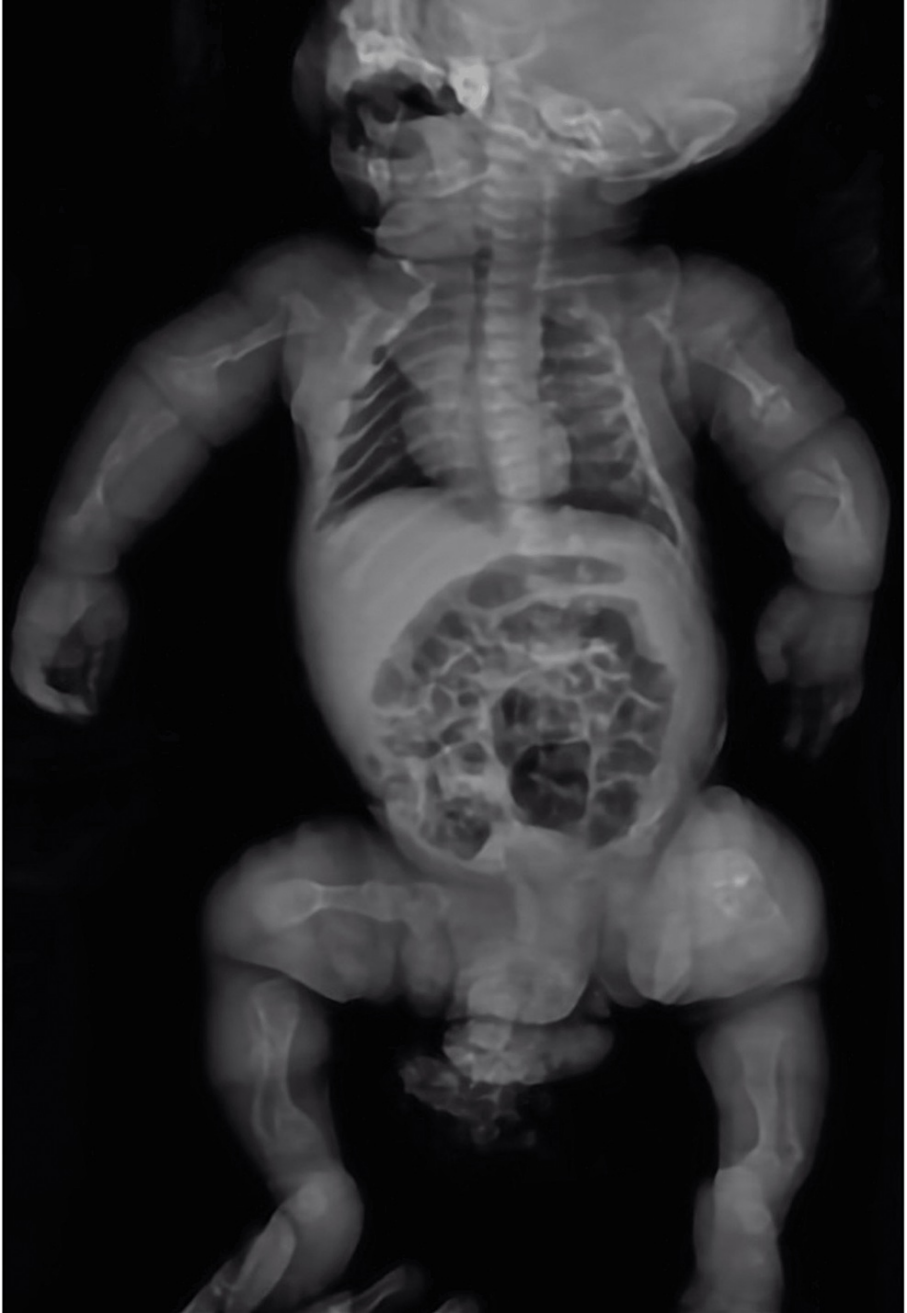
The patient at one month of age. Whole body X-ray showing multiple fractures in the long bones of the extremities causing deformity by shortening and bowing. Some of the fractures demonstrate signs of pseudarthrosis and some other prominent bony callus and angulation. The ribs exhibit posterolateral thinning and widening at the costochondral junctions (beaded ribs). Diffuse deficient mineralization is seen throughout the skeleton.

A male patient, with healthy and non-consanguineous parents, underwent an obstetric ultrasound during the 24th week of gestation, which documented significant shortening of the upper and lower extremities without associated fracture or deformity; the anteroposterior diameter of the thorax and the head circumference were found normal for gestational age. At birth at 38 weeks, multiple fractures of the upper and lower extremities, including the skull, were observed (Figure 2).

Phenotype

**Figure 3:**
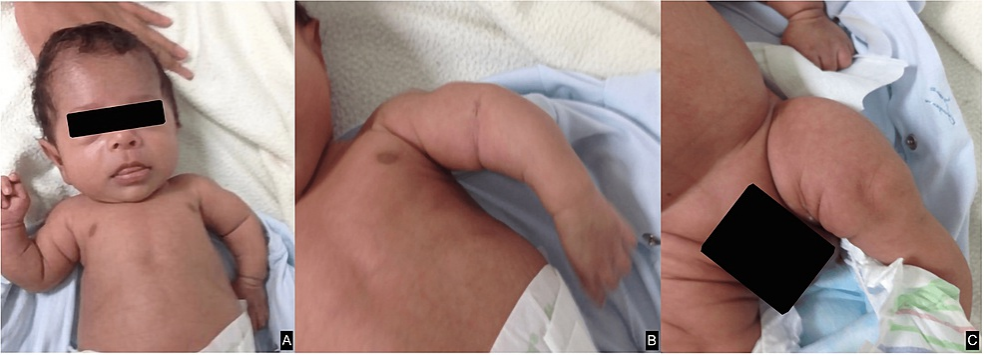
Clinical findings during the evaluation given by the genetics team. (A) Deformity in upper limbs given by shortening. (B) Micromelia of the upper limb and angular deformity. (C) Lower limb micromelia.

At one month and 15 days of age, the patient is evaluated by the genetics team. During the physical examination, they report a normocephalic skull with a wide forehead, blue sclerae, small nose, long nasolabial fold, and low implantation of the ears, as well as micromelia of the upper and lower limbs with a left equinovarus foot (Figure 3). His limbs showed fractures that have previously been reported by diagnostic images. Due to all these findings, they suggest a clinical case compatible with type II OI. Specialized care is suggested to minimize the risk of fractures and also continued regular follow-ups with pediatric orthopedics, pediatrics, and genetics services.

The diagnosis was made with the prenatal imaging findings and the postnatal clinical findings. Genetic sequencing is not presented due to the parents’ dissent of the examination. From the first year of life up to four years of age, the patient presented 19 more cases of fractures with a predominance of the upper limbs. Treatment with pamidronic acid was given at two years of age until 2019, followed by the start of zoledronic acid treatment until today, resulting favorably as there was a significant decrease in the number of fractures: two at the age of five and one at the age of six. In 2021, osteosynthesis with a telescopic nail of the right femur was done, this being his first surgical intervention. Currently, the patient is seven years old and presents a normal neurocognitive development, with an evident delay of growth in stature due to deformity secondary to shortening of the limbs due to fractures in the extremities.

Case 3

**Figure 4:**
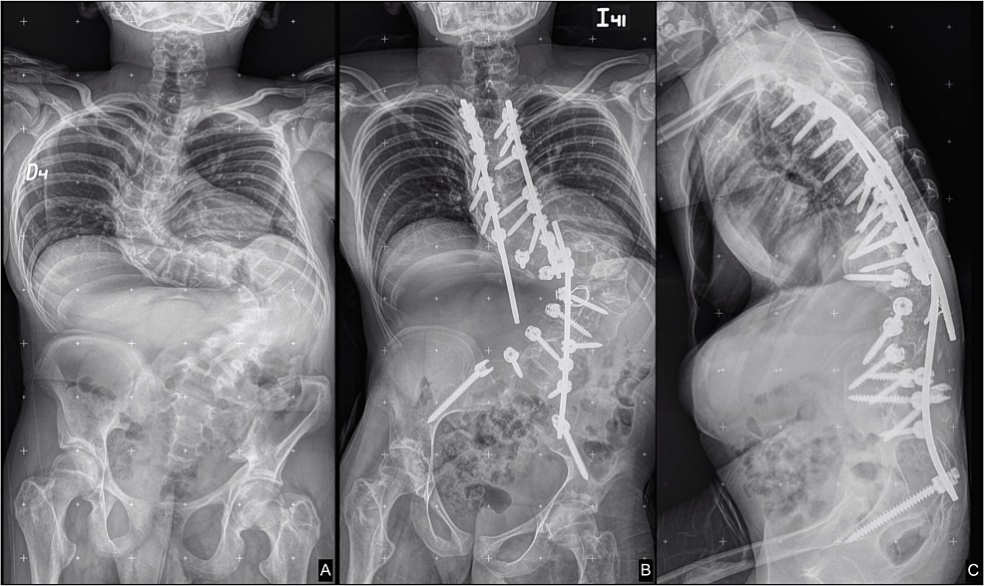
The patient at the age of 22. (A) Preoperative and (B-C) postoperative spinal X-rays showing severe left thoracolumbar kyphoscoliosis. The double contour of the femoral heads, iliac, and acetabula bones resembles the typical “bone-in-bone” posttreatment appearance. Also, the deformity of the rib cage is noted due to a substantial pectus excavatum.

A male patient product of the first pregnancy of non-consanguineous parents was born at 28 weeks via cesarean with acute fetal distress due to the mother’s preeclampsia. Being two and a half years old, fractures of the right tibia and femur were detected without any apparent trauma. At 10 years of age, he was seen for the first time by a genetics service that ordered a karyotype that was normal with a 46 XY, and no more complementary studies were ordered at the moment. A full spine X-ray was ordered, which documented severe scoliosis. Treatment in this patient was first started at five years of age with bisphosphonates. After the age of 20 years, the patient underwent teriparatide treatment and achieved an adequate response. Scoliosis surgery was performed without any complications (Figure 4).

Phenotype

At 19 years of age, the patient is once again seen by the genetics department who described the physical examination as a patient with a blue sclera, low implantation of the ears, and short neck. These, coupled with a medical history of pathological fractures without trauma and severe scoliosis, pointed to the clinical diagnosis of type I OI.

Case 4

A male patient, the first child of a 28-year-old mother, was born by vaginal delivery at 36 weeks without any difficulties or a need for a long hospital stay. The first nontraumatic fracture was detected in the left femur when the patient was 10 years old. Subsequently, other fractures were detected in the left tibia at age of 11 and in the left femur at age of 12.

Phenotype

**Figure 5:**
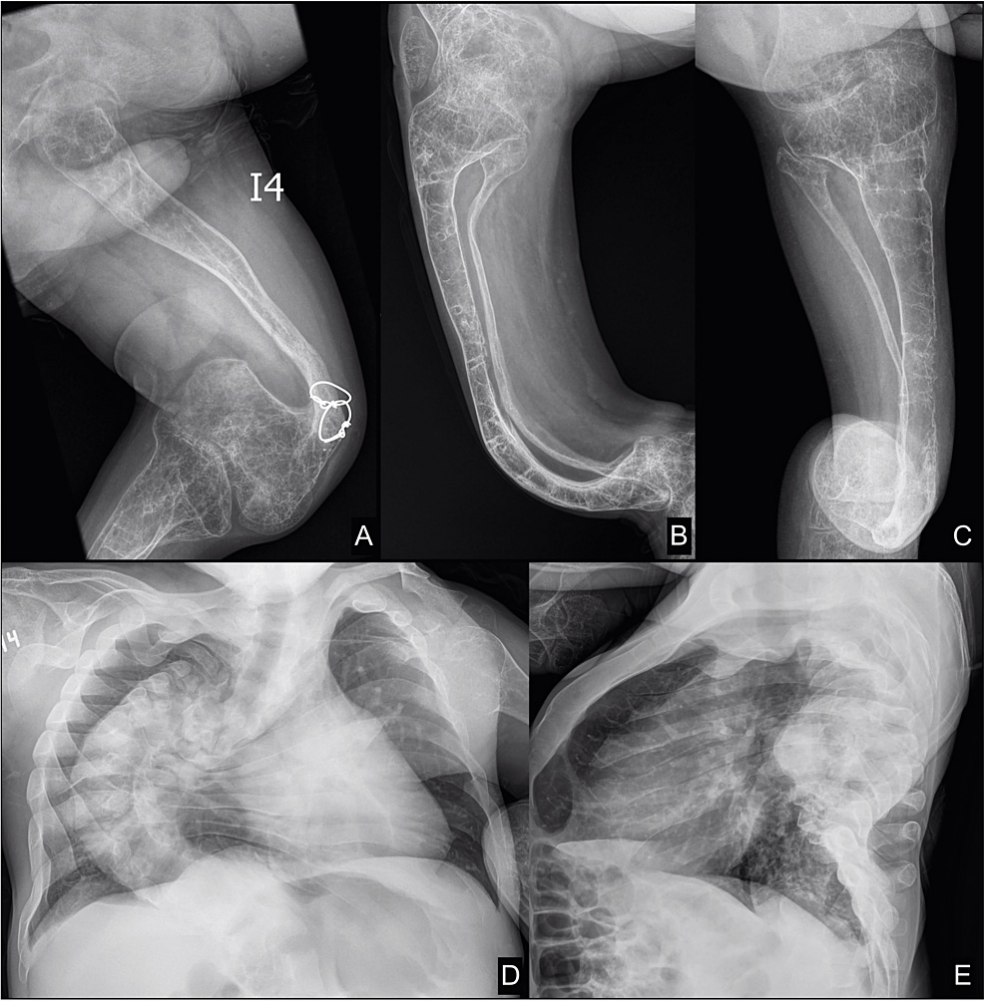
The patient at the age of 26. X-rays of the lower limbs, (A) left femur and (B-C) right leg, showing cortical bone thinning and excessive trabecular bone transparency due to osteopenia, as well as marked bowing (bending) of both diaphyses of the femur, tibia, and fibula with sequelae of previous fractures and operative intervention. (D-E) Chest X-ray reveals thoracic scoliosis and rib cage deformity due to pectus carinatum.

The genetics department described the physical examination as a patient with normal implantation of ears, pectus carinatum, and visible bowed legs (genu varum). These point to the clinical diagnosis of type II OI, which is supported by the medical history of pathological fractures without trauma and severely bowed legs. A continuous follow-up of the patient with current radiographs was carried out, which shows osteopenia, marked bowing of both diaphyses of the femur, and thoracic scoliosis (Figure 5).

## Discussion

Skeletal dysplasia, also known as osteochondrodysplasia, is the name given to a heterogeneous group of diseases that comprise abnormalities in the bone and cartilage due to genetic mutations. Their incidence is 1/5,000 live births and may occur in isolation or as a group of congenital defects, such as structural malformations, chromosomal pathologies, or polymalformative syndrome. More than 350 well-characterized skeletal dysplasias have been considered. These are classified as lethal, non-lethal, or variable lethality. Lethality can be assessed prenatally based on molecular and ultrasound parameters. Nevertheless, it is important to keep in mind that even though these mortality parameters exist, some patients may course with severe anomalies and still have a long survival rate. Furthermore, the definitive evaluation of lethality is given at birth [8,9].

Thanatophoric dysplasia, achondrogenesis II, and hypochondrogenesis are the most common lethal skeletal dysplasias. Regarding the non-lethal ones or the ones of variable lethality, there are OI, congenital spondyloepiphyseal dysplasia, and heterozygous achondroplasia [8,9].

OI includes a heterogeneous group of skeletal and connective tissue abnormalities characterized by the mutation of collagen genes, mainly the *COL1A1* and *COL1A2* type, with a subsequent decrease of bone mass and bone elasticity. This leads to a lower fracture point in comparison with a healthy bone, generating secondary joint instability. This fragility and constant healing fractures lead to angular deformities of the long bones [7-11].

There are different clinical presentations of OI. Secondary to the heterogeneity of its mutations, which leads to high variability in symptoms, this gave rise to the creation of Sillence’s (1979) initial classification. This classification groups them into categories I-IV, all of them considered autosomal dominant, and an X-linked variant in the type I presentation was added later (2013). Moreover, due to genetic and radiological advances, new types have recently been recognized, and the classification has been extended, including types V-XVIII, being types I-V predominantly autosomal dominant and the rest from VI to XVIII considered to be autosomal recessive [5-7,10].

The International Skeletal Dysplasia Society (2009) also suggested another classification that maintains the foundations of Sillence’s classification regarding severity and presentation. Including types I-V and avoiding involving genetic findings are essential and thus facilitate the clinical recognition of the disease [2].

Regarding the diagnosis of OI, it is mainly clinical and radiological. It can be made prenatally or postnatally. The clinical manifestations are variable, from mild forms to severe presentations, with the most distinctive characteristic being bone fragility, vulnerability to fracture due to minimal trauma or even its absence, and deformity or low growth. Extraskeletal manifestations include joint hypermobility, dentinogenesis imperfecta, blue sclera, and hearing loss. Muscle weakness and pulmonary and cardiovascular complications are rare. Frequently, it can be difficult to relate a case within a particular type of OI, so classification according to severity and signs found is still used [11].

During gestation, through the use of ultrasonography in the second and third trimester, osteoarticular changes can be observed that evidence the alteration of bone formation during gestation such as multiple fractures, osteopenia, demineralization, or even the loss of articular segment. From birth, the use of radiography is the most useful tool to demonstrate changes in the skeleton, where fractures are the most common [11].

The main radiographic features are osteopenia, bone fractures, and bone deformities. Radiographs are the most cost-effective method and usually reveal the thinning of the cortical bone and excessive transparency of the trabecular bone, as well as fractures that in many cases may be old. Other diagnostic tools used are bone density studies with bone densitometry by dual-energy X-ray absorptiometry (DEXA), which makes it possible to accurately determine the degree of bone demineralization. Serum calcium and phosphorus and skin and bone biopsy to study collagen tissue are also useful to guide the diagnosis; however, in some places, they are not widely used due to their high cost [12,13].

Once the diagnosis of OI has been made, treatment consists in providing a multidisciplinary approach, with the participation of different specialties such as neonatology, pediatrics, genetics, pediatric orthopedics, nutritionists, dental expertise, physical medicine and rehabilitation, and psychology in order to accomplish the main objective, which is to decrease the incidence of fractures and deformity, as well as to improve growth, pain, and the functionality of the patient. Within the natural history of OI, physical therapy and rehabilitation are purposely focused on maximizing gross motor function, especially during infancy with the increase of muscle strength in relation to mobility. Orthopedic surgery is the key treatment of OI because of its impact on life expectancy. This consists in performing long bone osteotomies and the placement of intramedullary nails to correct bone deformity, avoid fractures, and increase function [4,14,15].

Within pharmacologic treatment, there is a wide variety of options; the most used treatment is based on oral medication with bisphosphonates; within this group, there is pamidronate, alendronate, risedronate, neridronate, and olpadronate. This group works in the bone as an antiresorptive, by improving vertebral geometry, mainly by increasing the cortical and trabecular width of the bone. Despite the adequate result obtained in patients with OI, the consumption of bisphosphonates is currently questioned given that it has not achieved a significant decrease in long bone fractures [15-18].

Similarly, receptor activator of nuclear factor kappa Β ligand (RANK-L) inhibitors such as denosumab are also used. This is a monoclonal antibody that links to RANK-L and prevents the activation of receptor activator of nuclear factor kappa Β (RANK), it inhibits the function of the osteoclast and decreases bone resorption in cortical and trabecular bone. Cathepsin K inhibitors are also used, as it is expressed in osteoclasts, by inhibiting cathepsin K, which decreases bone resorption [1,4,14-16,19]. There are other pharmacologic treatments used in OI patients such as growth hormone therapy, anabolic treatments, and a new approach, which is the transforming growth factor B antibody [18].

Respecting the treatment with osteo-anabolic therapies, there is teriparatide, which is a parathyroid hormone (PTH) analog, and abaloparatide, a synthetic analog of PTH-related peptide. Teriparatide and abaloparatide promote osteoblast differentiation and bone formation. Its use is restricted in the pediatric population due to the higher risk of developing osteosarcoma. As in case 3 of this case series, the patient was managed during the pediatric age with bisphosphonates, and after the age of 20, therapy with teriparatide was started [18].

A meta-analyses carried out by Shi and colleagues has shown that bisphosphonates are associated with a moderate decrease in fracture rates; nevertheless, in a double-blind randomized trial, teriparatide treatment has been shown to be superior to risedronate (taken orally) in preventing vertebral fractures [20-22].

Regarding the cases analyzed in this review, which consist of one case of type I OI, two cases of type II OI, and one case of type III OI, two of them were diagnosed during pregnancy, and the other two were diagnosed after birth. One of the cases underwent genomic sequencing, but the other cases were unable to access the test. Nevertheless, a clinical and immunological diagnosis was obtained by the signs and symptoms that were typical of OI, allowing the right classification. All cases were treated with multidisciplinary and individualized management, in which the orthopedic department played an important role since the sequelae of the patients derive mainly from fractures causing deformity and limitation of functionality. In addition, the follow-up of these patients was performed by other specialties as advised in the literature. It is important to highlight that these patients have not presented alterations in cognitive or neurodevelopmental functions; however, the evolution of their bone disease has been torpid.

Given that clinical variants of OI exist, other findings suggested by different researchers are summarized in Table 1 [5-7,10,11,17,18,23].

**Table 1:** Molecular types and clinical classification of osteogenesis imperfecta (OI) in accordance with its clinical presentation, severity, inheritance, and mutations. Mutation group: (A) alteration in the synthesis and processing of collagen, (B) defect in mineralization, (C) abnormal modification of collagen, (D) compromise in processing and folding of collagen, and (E) alteration of the development and function of the osteoblasts. AD, autosomal dominant; AR, autosomal recessive Source: [5-7,10,11,17,18,23]

Mutation group	Molecular type	Clinical presentation	Severity	Inheritance	Associated mutations	Clinical classification
A	I, 85%-90%	Normal stature or slightly decreased, blue sclerae, hearing loss, and some fractures	Slight and nondeforming	AD	*COL1A1* (which leads to a decreased production of collagen type I), *COL1A2*, and *BMP1*	Type I
	II, 85%-90%	Blue-gray sclerae, arched long bones, skull malformation, multiple rib fractures and long bone fractures present at birth, and alteration in pulmonary capacity due to thoracic restriction	Lethal in the prenatal period and severe in different molecular subtypes	AD and rarely AR	*COL1A1*, *COL1A2*, and *CREB3L1*	Type II
	III	White sclerae, multiple fractures with deformity and short stature, characteristic popcorn calcifications, extremely short stature, and triangular face	Severe	AD/AR	*COL1A1*, *COL1A2*, *CRTAP*, *P3H1*, *PPIB*, *FKBP10*, *SERPINH1*, *PLOD2*, *BMP1*, *SEC24D*, *P4HB*, *SERPINF1*, and *SP7*	Type III
	IV	White sclerae, slight bone deformation, and short stature	Moderate	AD/AR	*COL1A1*, *COL1A2*, *CRTAP*, *FKBP10*, *SERPINH1*, *PLOD2*, *BMP1*, *SPARC*, *TMEM38B*, *SEC24D*, *SERPINF1*, and *WNT1*	Type IV
B	V	Calcification of the interosseous membrane of the forearm and a hyperplastic callus, white sclerae, and stature variability	Moderate	AD	IFITM5	Type V
	VI	White sclerae and fish scale pattern in iliac crest biopsies. Histology: osteoid formation due to mineralization violation	Moderate to severe	AR	SERPINF1	Type VI
C	VII	White sclerae, rhizomelia, long bone deformities, proximal shortening of the limbs, and coxa vara deformities, accompanied by dentinogenesis and normal sclerae	Moderate	AR	CRTAP	Types II, III, and IV
	VIII	White sclerae, platyspondyly, and clinically similar to type II OI. Diagnosis is made perinatally. The characteristics of bone deformities are being severe, white sclerae, and normal dentinogenesis	Severe and lethal in the perinatal period	AR	*LEPRE1* also known as *P3H1*	Types II and III
	IX	Skeletal deformity, white sclerae, and short stature	Severe	AR	PPIB	Types II, III, and IV
D	X	Blue sclerae, imperfect dentinogenesis, cutaneous anomalies, inguinal hernia, and nephrolithiasis	Severe	AR	*SERPINH1* and *FKBP10*	Type III
	XI	Blue-gray sclerae and congenital articular contractures	Moderate to severe	AR	*SERPINH1* and *FKBP10* genes	Types III and IV
	XII	Skeletal deformities and umbilical hernia	Moderate to severe	AR	*PLOD2* and *BMP1* genes lead to incomplete type XII osteogenesis	Type IV
E	XIII	Skeletal deformity and facial hypoplasia	Severe	AR	SP7	Type III
	XIV	Bone deformity and sclerae that can be blue or normal	Severe	AR	TMEM38B	Type III
	XV	Neurological deficit	Severe	AR/AD	WNT1	Types III and IV
	XVI	Perinatal features and long bone fractures	Severe	AR	CREB3L1	Type III
	XVII	Bone fragility	Progressive and severe	AR	SPARC	Type III
	XVIII	Developmental delay, dysmorphic features, scoliosis, and pectoral deformities	Moderate to severe	AR	FAM64A	Type III

Survival depends on the type of OI. In most cases, the degree of severity is high with great limitations and complications. However, despite the high mortality, many case reports have shown prolonged survival as in the cases presented in this manuscript. It is important to highlight that OI is a disease without a cure and that it is of utmost importance to provide an appropriate approach to care that allows early intervention with the necessary medications and surgical procedures that will result in the comprehensive management of the disease to avoid progression to deformities.

## Conclusions

OI is a disease with multiple clinical findings and genetic variations. An early diagnosis is important; it allows a comprehensive and multidisciplinary approach to the management of the patient. On the contrary, the consequent fractures and bone deformities will decrease the quality of life of these patients.
